# Repair of Tortuous Distal Aortic Arch Aneurysm in a 1-Year-Old Girl With PHACE Syndrome

**DOI:** 10.1016/j.atssr.2023.02.004

**Published:** 2023-02-20

**Authors:** Koji Okamoto, Yoshie Ochiai, Kunihiko Joo, Yoshiyuki Yamashita, Yusuke Nakata, Jun Muneuchi, Shigehiko Tokunaga

**Affiliations:** 1 Department of Cardiovascular Surgery, Japan Community Healthcare Organization (JCHO) Kyushu Hospital, Kitakyushu-city, Japan; 2 Department of Pediatric Cardiology, Japan Community Healthcare Organization (JCHO) Kyushu Hospital, Kitakyushu-city, Japan

## Abstract

Herein, we describe a 21-month-old girl with PHACE syndrome (posterior fossa hemangiomas, arterial lesions, cardiac anomalies/coarctation of the aorta, and eye anomalies) who presented with a tortuous extensive aortic arch aneurysm. As the maximum short diameter of the distal aortic arch aneurysm expanded rapidly from 21 mm to 25 mm in only 5 months, we performed extensive aortic arch reconstruction with interposition graft replacement through a left thoracotomy under partial cardiopulmonary bypass.

The aortic arch anomalies observed in PHACE syndrome (posterior fossa hemangiomas, arterial lesions, cardiac anomalies/coarctation of the aorta, and eye anomalies) are complex and often involve long segments of the transverse aortic arch and descending thoracic aorta.^1^^,^^2^ After preoperative imaging for aortic and cerebrovascular arterial anomalies, extensive arch reconstruction by nonnative tissue techniques is often required, even in infants with PHACE syndrome. We report a tortuous distal aortic arch aneurysm in a 21-month-old girl with PHACE syndrome.

At 1 month of age, the patient was diagnosed with a hemangioma in the left auricle, which extended to the entire left side of the head. At 4 months, she was diagnosed with PHACE syndrome after detection of hemangioma of the head, a tortuous left internal carotid artery, and a tortuous distal aortic arch aneurysm extending to the descending aorta. Aspirin and β-blocker were initiated to prevent arterial thrombosis and to decrease the size of the facial hemangioma and aneurysm, and at 18 months, the size of the facial hemangioma had decreased. However, 5 months later, contrast-enhanced computed tomography revealed that the maximum short diameter of the distal aortic arch aneurysm had enlarged from 21 mm to 25 mm. As shown in Figure 1, a bizarre saccular aneurysm developed after the origin of the left vertebral artery and tortuously extended down to the descending thoracic aorta at the level of the fifth thoracic vertebra. Magnetic resonance angiography showed a tortuous left internal carotid artery and a complete circle of Willis. Echocardiography revealed normal intracardiac anatomy, and blood pressure was similar in both upper and lower limbs preoperatively.

**Figure 1:**
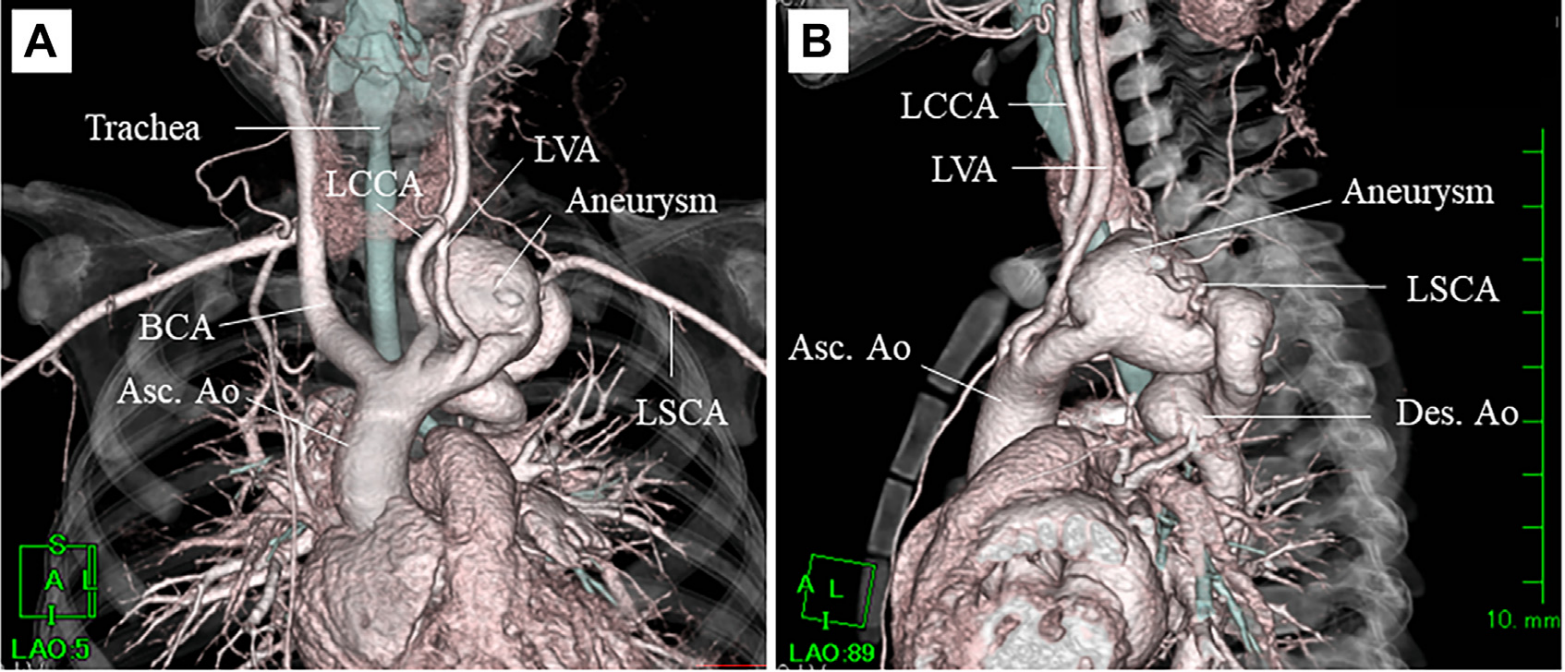
Preoperative 3-dimensional computed tomography revealed a tortuous aortic arch aneurysm from the distal aortic arch to the descending aorta (Des. Ao). (A) Frontal view. (B) Left lateral view. (Asc. Ao, ascending aorta; BCA, brachiocephalic artery; LCCA, left common carotid artery; LSCA, left subclavian artery; LVA, left vertebral artery.)

At 21 months of age and a weight of 11 kg, operation was required to prevent aortic rupture. As this case involved an atypical tortuous saccular aneurysm requiring prolonged cross-clamp time, we performed aortic arch reconstruction with interposition graft replacement through a left thoracotomy under partial cardiopulmonary bypass (CPB) between the main pulmonary artery and the descending thoracic aorta. A thoracotomy was performed at the left posterior fourth interspace. Thereafter, the pericardium was incised anterior to the phrenic nerve; the ligament of ductus arteriosus was divided, and the ascending aorta, left carotid artery, retroaortic innominate vein, and left vertebral artery were dissected. After heparinization, we inserted an arterial cannula into the lowest convenient location in the descending aorta and a venous drainage cannula into the main pulmonary artery to start the partial CPB. Partial CPB entailed the use of a roller pump with an oxygenator, maintaining the pump flow rate at 40% to 50% of the maximal flow rate (2.8 L/min per m^2^). A proximal clamp was applied to the distal arch just after the origin of the left carotid artery with occlusion of the left vertebral artery by a small clamp, and the transverse aorta was divided just proximal to the aneurysm. A 12-mm-diameter Gore-Tex vascular graft (W. L. Gore & Associates) was then sutured to the distal arch with continuous 5-0 Prolene sutures (Ethicon). Before performing the distal anastomosis, we opened the aneurysm and verified that there were no inflow arterial vessels. The wall of the excised aortic aneurysm was sent for histopathologic examination, and the left subclavian artery was sacrificed from the aneurysmal wall. To match the diameter of the anastomotic part of the descending aorta to the size of the 12-mm Gore-Tex graft, we made a bevel incision in the descending aorta and anastomosed the graft to the descending aorta with 6-0 Prolene sutures. Air was removed from the distal anastomosis site by removing the clamp at the descending thoracic aorta, followed by slow removal of the clamp at the Gore-Tex graft. Decannulation was performed after heparin reversal.

The patient showed no paraplegia or phrenic nerve palsy postoperatively. Endotracheal extubation was achieved on postoperative day 1. Postoperative computed tomography confirmed the smooth shape of the interposition graft, as shown in Figure 2, and the patient was discharged on postoperative day 24. Histopathologic evaluation of the aneurysmal wall revealed irregularly distributed elastic tissue, smooth muscle, and myointimal cells, which are characteristic of PHACE syndrome (Figure 3).

**Figure 2:**
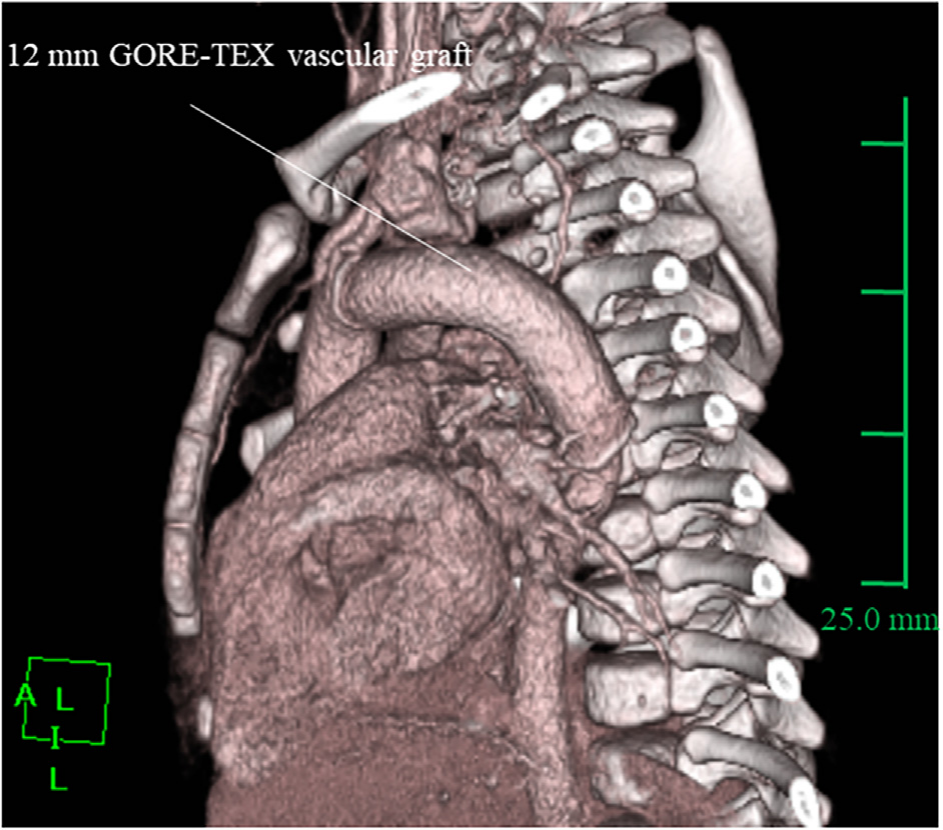
Postoperative 3-dimensional computed tomography image showing smooth interposition graft.

**Figure 3:**
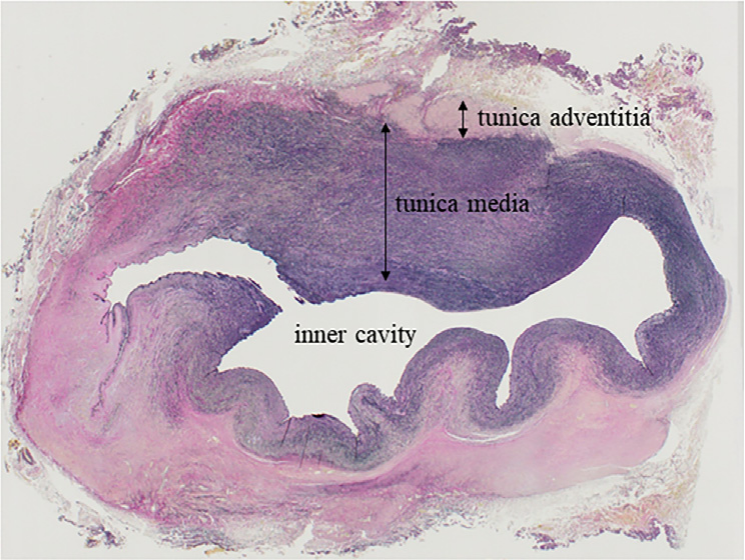
Microscopic examination of the aneurysmal wall demonstrated a dysplastic aorta with irregularly distributed elastic tissue, smooth muscle, and myointimal cells (elastica van Gieson stain, magnification ×12.5).

At 6.5 years postoperatively, the patient is asymptomatic and is not taking cardiac medications. There is no gradient across the arch to the descending aorta by Doppler echocardiography.

## Comment

Vascular abnormalities in PHACE syndrome include formation of an aneurysm with dilation and narrowing of the artery from the aortic arch to the descending thoracic aorta with cerebrovascular arterial dysplasia.^1^, ^2^, ^3^, ^4^ However, long-segment saccular aneurysms with widespread aortic tortuosity extending into the descending aorta are rare. In our case, we considered that the fragile aneurysm wall adhered to the surrounding tissue, which would lead to a longer cross-clamp time. Therefore, we ruled out an autologous tissue reconstruction like extensive dissection and end-to-end anastomosis and selected placement of the largest possible interposition graft for her physique under partial CPB through left thoracotomy.^5^, ^6^, ^7^

Backer and colleagues^5^ and Fiore and coworkers^6^ reported the use of left atrium–to–descending aorta CPB to protect the spinal cord in patients with mild and complex coarctation. In our institute, the preferred location of the venous drainage is in the main pulmonary artery instead of the left atrium because cannulation into the left atrium is cumbersome and air embolism is possible. In addition, this partial CPB for lower body and spinal cord perfusion through a left thoracotomy is a safe technique that allows longer cross-clamp times. Notably, 2 patients with 10-mm and 8-mm grafts interposed at 2 months and 5 days, respectively, required extra-anatomic bypass grafting using CPB at 11 years of age and 8 years of age, respectively, with a 20-mm graft.^2^ Therefore, it is inevitable that the 12-mm interposition graft will become progressively obstructive in our patient as she grows, and she may require an extra-anatomic bypass and reintervention through median sternotomy. Long-term follow-up is mandatory to determine better optical management strategies.

In conclusion, we encountered a rare case of a tortuous, long-segment distal aortic arch aneurysm in a 21-month-old girl with PHACE syndrome. Extensive graft interposition through a left thoracotomy approach was achieved under partial CPB between the main pulmonary artery and descending aorta.
